# *QuickProcess2*: an integrated package for crystallography data visualization, processing and management at the GM/CA Structural Biology Facility

**DOI:** 10.1107/S1600576726008289

**Published:** 2026-09-30

**Authors:** Qingping Xu, Mark C. Hilgart, Nagarajan Venugopalan, David Kissick, Michael Becker, Shenglan Xu, Oleg Makarov, Sergey Stepanov, Janet L. Smith, Robert F. Fischetti

**Affiliations:** a GM/CA, Advanced Photon Source Argonne National Laboratory 9700 South Cass Avenue Lemont Illinois60439 USA; b Life Sciences Institute and Department of Biological Chemistry University of Michigan Ann Arbor Michigan48109 USA; DESY, Hamburg, Germany

**Keywords:** macromolecular crystallography, serial synchrotron crystallography, diffraction image viewer, automated data processing

## Abstract

The rapid evolution of macromolecular crystallography towards high-frame-rate detectors and complex serial data collection strategies requires better integration of traditional software tools and the development of new solutions. Managing data flow, assessing quality in real time and merging results from thousands of crystals – as required by synchrotron serial crystallography – present significant challenges for beamline operations. Existing solutions often fragment the workflow into disconnected steps: visualization, processing and experiment data management. We present *QuickProcess2* (*QP2*), an open-source package designed to unify these tasks into a cohesive system. Built on a client–server architecture using Python, Redis and HDF5, and developed using AI-assisted coding, *QP2* integrates a low-latency ‘Live’ image viewer with an automated, context-aware data processing engine. Key features include native support for multiple datasets that facilitates the handling of both standard rotation and serial crystallography experiments; a flexible plugin-based architecture for integrating processing suites, such as *XDS* and *CrystFEL*; and persistent database tracking of all experiment parameters and results. The package offers user-friendly visualization and processing features, while also providing dedicated tools for beamline staff. By bridging the gap between data collection and reduction, *QP2* aims to streamline beamline operations and support efficient handling of high-throughput data.

## Abstract 2

*QuickProcess2* (*QP2*) unifies real-time image visualization, automated multi-pipeline data processing and experiment management into a single open-source platform for synchrotron macromolecular crystallography beamlines, enabling live quality assessment and concurrent data reduction for both standard rotation and serial crystallography experiments.

## 1. Introduction

The deployment of high-frame-rate pixel array detectors and automated sample delivery systems at synchrotron beamlines has shifted structural biology into a high-throughput data regime. Next-generation synchrotron sources – such as the upgraded Advanced Photon Source (APS), which provides beams much smaller and up to 500 times brighter than its predecessor – now deliver an unprecedented photon flux. Pixel array detectors capable of framing rates up to 1000 Hz enable high-speed data acquisition, while beamline automation technologies provide automated sample handling and data collection. These advancements have enabled synchrotron serial crystallography (SSX), where data from hundreds to thousands of microcrystals are merged to solve a single structure. Such conditions require software infrastructures capable of processing multi-gigabyte data streams in real time to minimize latency during volume-intensive SSX experiments. The ability to visualize data in real time, trigger automated processing pipelines and rapidly assess diffraction quality has become a critical requirement for optimizing user experience and ensuring efficient beamtime utilization.

While software solutions for individual aspects of crystallography are well established – with programs like *adxv* (Arvai, 2012 ▸) for visualization, *MOSFLM* (Leslie & Powell, 2007 ▸), *XDS* (Kabsch, 2010 ▸), *DIALS* (Winter *et al.*, 2018 ▸) and *xia2* (Winter, 2010 ▸) for data processing, and *CCP4* (Winn *et al.*, 2011 ▸), *SHELX* (Sheldrick, 2008 ▸) and *PHENIX* (Adams *et al.*, 2010 ▸) for structure solution and analysis – the overall landscape remains fragmented. Visualization tools that are suitable for beamline operations often operate in isolation. For example, legacy tools like *adxv* have set the standard for visualization and offer real-time monitoring via socket interfaces, yet they are primarily designed for single-dataset inspection. They often lack the integrated context required to manage the data flow or processing results of multi-dataset serial crystallography experiments. Similarly, modern tools like *dials.image_viewer* (Winter *et al.*, 2018 ▸) and *Albula* (DECTRIS, https://www.dectris.com) offer specific capabilities – such as detailed pixel analysis or vendor-specific optimizations – but often operate as standalone applications, or as part of large data analysis suites that are not designed for real-time monitoring or integration with processing pipelines at the beamlines.

Thus, users often need to juggle multiple independent applications: one for viewing images, another for submitting processing jobs, and a third for tracking results or managing experiment metadata. This context-switching can be inefficient, particularly during complex serial data collection. Consequently, beamlines frequently build custom integrated environments to bridge the gap between data collection and reduction. Specifically, such a platform must provide speed and streaming capabilities to render megapixel images at high frame rates from data streams. It requires deeper integration, allowing a direct transition from visualization to processing – moving from inspecting a diffraction spot to indexing a lattice with a single click. The software must possess context awareness, utilizing experiment metadata (energy, beam center, distance) to auto-configure pipelines, and ensure operational simplicity by bridging the gap from detector to structure and removing the need to navigate complex directory trees. Crucially, for serial crystallography experiments, the system must natively support multi-dataset management, handling ‘experiment groups’ of many crystals simultaneously. Additionally, it should enable users to play an active role in inspecting and processing the data, rather than treating them as a black box.

Various software packages have been developed to address these needs, each with its own strengths and weaknesses. For example, *SynchWeb* (Fisher *et al.*, 2015 ▸) serves as a modern, responsive web interface for the ISPyB (Delagenière *et al.*, 2011 ▸) database, streamlining sample registration, experiment tracking and the visualization of automated processing results for remote monitoring. It consolidates workflow management – from sample shipping to structure solution – into a single platform accessible via mobile devices. However, its reliance on a complex, facility-wide database infrastructure can present a significant barrier to adoption for individual beamlines. Similarly, *MxLIVE* (Fodje *et al.*, 2012 ▸), developed at the Canadian Light Source, provides a web-based laboratory information management system (LIMS) that integrates with the *MxDC* data acquisition software to manage samples and monitor experiments remotely. While effective for monitoring and management, these systems operate primarily as portals to a database rather than high-performance, real-time workflows tightly integrated with the detector data stream. Such tight integration is essential for providing the immediate feedback and control required to effectively adapt to the demands of SSX.

At the National Institute of General Medical Sciences and National Cancer Institute Structural Biology Facility at the Advanced Photon Source (GM/CA@APS, or GM/CA), we previously developed basic data processing capabilities that were embedded in the data collection software (Pothineni *et al.*, 2014 ▸). We then developed the *QuickProcess* package with improved usability and additional capabilities. Here, we describe *QuickProcess2* (*QP2*), a package that unifies image visualization, preliminary data analysis, automated data processing and experiment management into a single cohesive system. *QP2* employs a client–server architecture using the Redis databases (https://redis.io/) for processing state persistence and rapid inter-process communication, and an SQL database for permanent metadata persistence, ensuring reliable operation in beamline environments. In the following sections, we describe the system architecture, the image viewer, and the automated processing infrastructure designed for both conventional rotation and serial crystallography data. The development was greatly accelerated and a working prototype was completed in 6 months by utilizing AI-assisted coding through large language model (LLM) APIs.

## 2. Results and discussion

### 2.1. Software architecture

**Figure 1:**
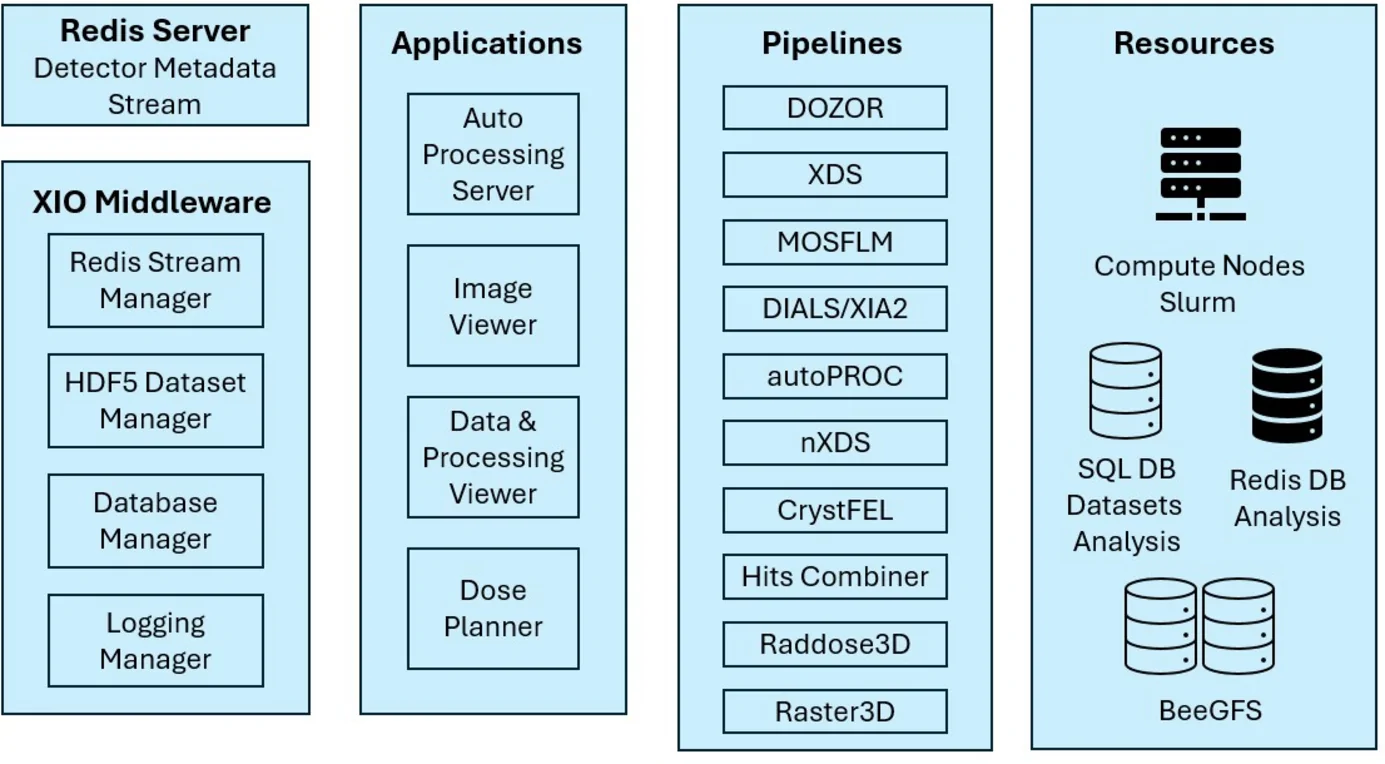
Diagram of *QP2* architecture. The platform integrates user-level applications, such as a high-performance image viewer and data and processing viewer, with a distributed processing backend. These are built on top of the xio library interacting with Redis and HDF5 files stored on a shared file system. Real-time communication and state management are handled via Redis. Processing pipelines (*nXDS*, *DOZORetc*.) are executed on *Slurm* clusters or local workstations, with results streamed back to the user interfaces for immediate visualization and decision-making.

*QP2* employs a modular client–server architecture (Fig. 1 ▸) designed to decouple visualization from heavy computational tasks while ensuring data integrity and responsiveness. Rather than directly interacting with GM/CA’s open-source data collection software (*PyBluIce*, https://www.gmca.aps.anl.gov/computing/jbluice-epics.html), the *QP2* image viewer and auto-data processing server listen to the detector events via a Redis stream that broadcasts metadata of ongoing frames, such as collection parameters, file locations, frame indices and timestamps. The core system is built on Python, utilizing *PyQt5* for the graphical user interface.

At the foundation lies the xio library, which abstracts all I/O operations. From the Redis message stream, the Redis stream manager reconstructs data collection events, records them with corresponding metadata in an SQL database and dispatches them to the appropriate handlers (image viewer or auto-processing). For each data series, an HDF5 manager extracts and stores metadata and builds a map of expected files. It periodically polls the shared file system for new files and emits status updates to applications like the image viewer and auto-processing server until the dataset is fully collected. The HDF5 manager is optimized to minimize the impact on the file system by skipping repeated checking of files that are confirmed to be on disk. A database manager is used to store dataset collection experiment metadata after each collection, as well as data processing results from various processing jobs. A package-level logger provides a centralized way to log all events and errors in a consistent manner.

*QP2* utilizes a tiered persistence strategy. Ephemeral data (processing states, temporary processing results for display) are stored in the memory or memory-backed Redis database, providing sub-millisecond latency essential for above 100 Hz data streams. Long-term experiment history, processing statistics and configurations are stored in an SQL database. Large processing files are stored on the network distributed file system (*e.g.* BeeGFS at GM/CA). This ensures that every processing job, whether automatic or manual, is fully reproducible.

### 2.2. Image Viewer

**Figure 2:**
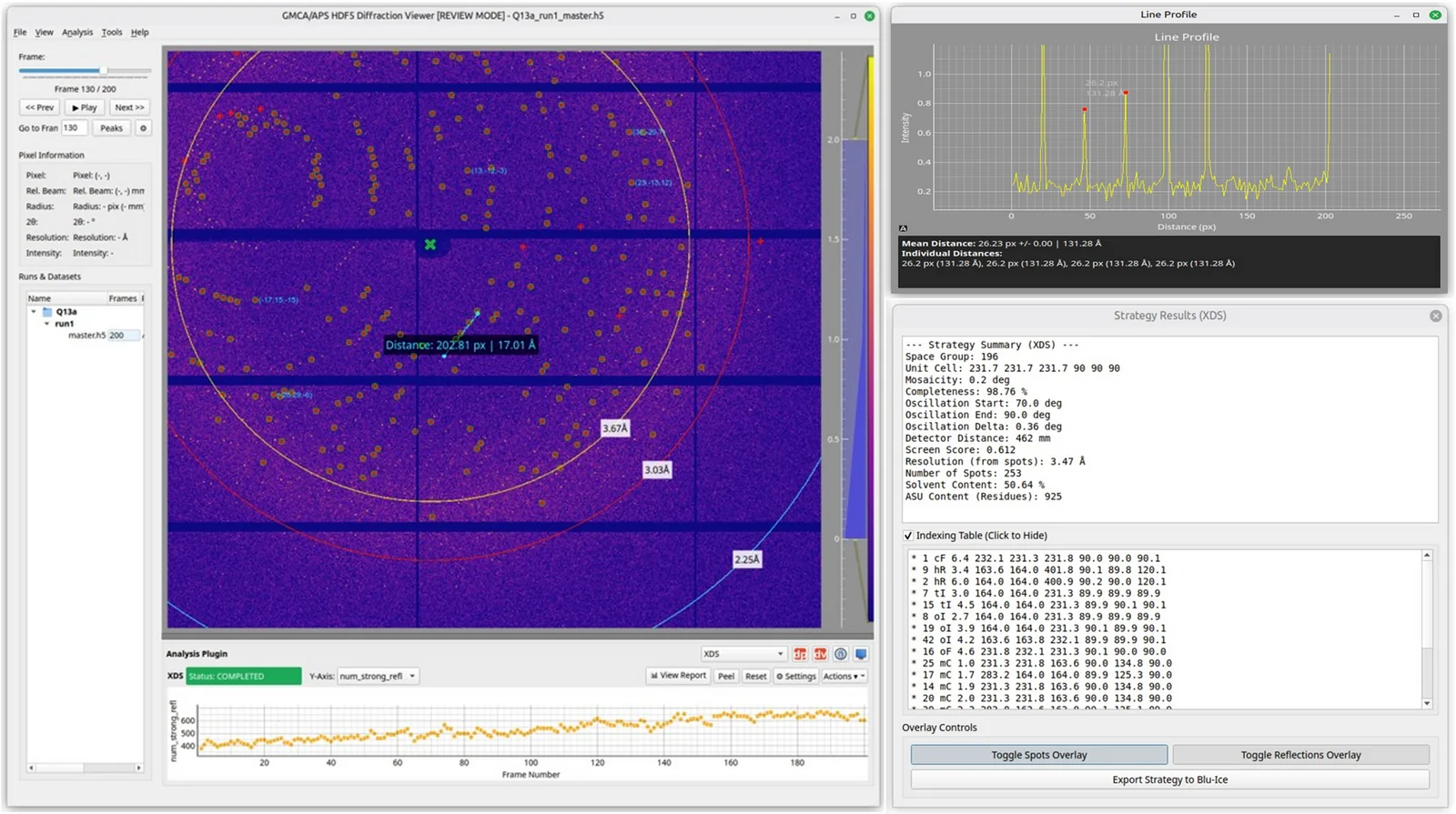
*QP2* Image Viewer interface. The primary viewport displays a diffraction pattern with overlaid resolution rings. A 1D intensity profile integration region is marked by a cyan dashed line, with the resulting plot shown in the upper-right panel. Processing integration is demonstrated via the overlay of observed spots (red crosses) and predicted reflections (green squares) from an *XDS* indexing job, with detailed indexing statistics and results presented in the lower-right panel. Beneath the main image, plugin subwindows display processing metrics, such as the number of strong spots versus frame index from the *XDS* processing job.

The Image Viewer (iv) makes use of the Redis stream manager and HDF5 manager (using the *h5py* library, https://www.h5py.org/) to keep track of incoming data and display them in real time (Fig. 2 ▸). It can also operate in a Review mode to explore existing HDF5 datasets. A critical requirement for the iv during beamline operations is rendering speed; the viewer can ingest and display data from detectors operating at frame rates exceeding 100 Hz by adaptively displaying selected frames. To handle the high data rates of modern pixel detectors (*e.g.* EIGER2 XE CdTe 16M), the viewer utilizes a highly optimized rendering pipeline based on *PyQtGraph* (Moore *et al.*, 2023 ▸). This enables smooth panning, zooming, a rich selection of colormaps and contrast adjustment of multi-megapixel images at reasonable frame rates. In cases where data arrive at a high rate, the viewer utilizes an adaptive playback mechanism to automatically skip frames during fast replays to keep up with the latest frames.

The iv was inspired by the best features of *adxv*; we implemented these core capabilities, such as detailed pixel information at mouse location, zoom to pixel value, 1D or 2D profile on region of interest, smart initial contrast selection, auto-contrast adjustment on zooming into specific regions, customizable resolution ring overlay, distance measurement on image, displaying image metadata *etc*. We advanced these diagnostic capabilities by natively projecting real-time processing results – such as predicted lattice reflections and spot integration masks – directly onto the active diffraction image. In addition to standard playback controls, we added a slider and a frame index field that allow users to navigate to any frame directly, making exploration of frames within a dataset significantly easier. For detailed inspection, the viewer offers a robust set of image analysis and manipulation tools. An ice-ring analysis tool detects and characterizes ice rings within the image, while on-the-fly summation allows users to sum multiple consecutive frames to enhance the signal-to-noise ratio of weak reflections. Real-time filtering, such as Poisson background subtraction and Gaussian smoothing, can be toggled to highlight specific features. Finally, an interactive mask tool facilitates the creation of custom geometric masks for processing inclusion or exclusion.

The iv implements a Python-based spot finder capable of detecting and annotating diffraction spots in real time during image playback. To achieve the necessary speed, it employs a cascading filtering strategy where each step dramatically reduces the problem size, thereby minimizing the overall computational load. The process begins with *scikit-image*’s (van der Walt *et al.*, 2014 ▸) peak_local_max algorithm, which rapidly identifies a broad set of initial peak candidates on the basis of local intensity maxima. These candidates are then systematically pruned through a series of lightweight filters: invalid detector regions are masked, spatial separation constraints are enforced and low-intensity peaks are discarded. This ensures that the most computationally intensive operations – specifically the Z-score (signal-to-noise ratio, SNR) calculation, which compares integrated intensity against a local annular background – are performed on only a small fraction of the original candidates. This hierarchical approach allows for robust detection in weak-signal regimes while maintaining high frame rates. Additional heuristics, including minimum pixel count thresholds and resolution-dependent analysis to reject ice-ring artifacts, ensure high fidelity. Detection parameters – such as SNR thresholds, spot size and background estimation radii – can be dynamically adjusted on the fly, providing immediate visual feedback on detection performance.

**Figure 3:**
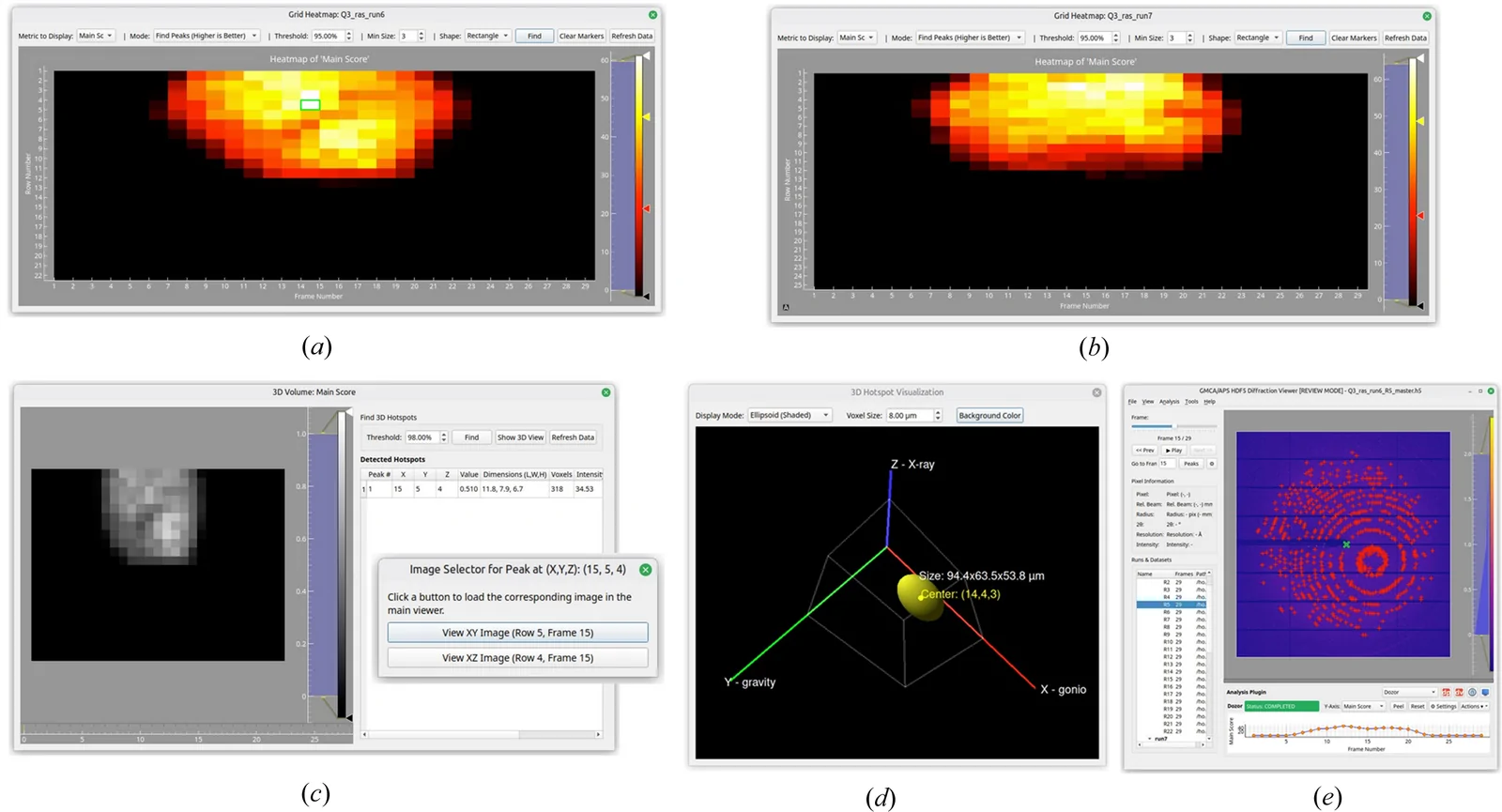
Crystal reconstruction via orthogonal X-ray raster scans. In this example, a test crystal was centered within the loop and scanned with a cell size of 8 × 8 µm (22 × 29 and 25 × 29 grids). (*a*) 2D diffraction heatmap from a raster scan at a goniometer setting of 0° (dark low *DOZOR* main score, bright high *DOZOR* main score). (*b*) 2D diffraction heatmap from the orthogonal scan at 90° (dark low *DOZOR* main score, bright high *DOZOR* main score). (*c*) Approximate 3D localization model generated by computing the tensor product of the 2D metric arrays. (*d*) Ellipsoidal fitting of the 3D crystal volume, where dimensions and principal orientation are estimated via eigenvalue analysis of the spatial covariance matrix. (*e*) Diffraction pattern collected at 0° at the calculated geometric center.

To facilitate the analysis of raster scans and grid-based data collections, the iv implements a dynamic 2D heatmap viewer. This module actively reconstructs the spatial relationship between grid points by parsing file metadata (rows/columns) and mapping them to a unified coordinate system. It allows users to visualize any scalar metric generated by the processing pipelines – such as score, spot count or Bragg spacing (resolution) – as a color-coded map. The viewer handles large datasets asynchronously, fetching results from the Redis state database or file system without blocking the user interface (UI). Integrated analysis tools include a hotspot finder that utilizes *scipy.ndimage* (Virtanen *et al.*, 2020 ▸) to detect clustered regions of high values (peaks) or low values (valleys) on the basis of percentile thresholds, automatically calculating the center of mass and orientation of crystal hits. Furthermore, the heatmap is fully interactive: clicking on any cell instantly navigates the main iv to the corresponding diffraction frame and overlays the specific analysis results (predicted spots, reflections) for that position, enabling rapid validation of processing outcomes. For samples that can be approximated as separable objects, an approximate 3D localization volume can be generated from the tensor product of two orthogonal 2D raster scans (*e.g.* at 0° and 90°; Fig. 3 ▸). Diffraction patterns for each cell are analyzed [*e.g.* by *DOZOR* (Zander *et al.*, 2015 ▸)], and a 3D scalar volume is generated by computing the tensor product of the resulting 2D metric arrays. A hotspot detection algorithm then segments high-intensity regions to identify individual crystals, estimating their center of mass, dimensions and principal orientation, which allows improved crystal centering for subsequent data collection. When beamline grid geometry is available, these reconstructed crystal positions can also be converted into motor coordinates for direct centering on the goniometer. Derived size and orientation parameters can also be input into the ‘Dose Planner’ (see Section 2.5 below) for sample-specific radiation damage modeling.

A core feature of the iv is its advanced dataset context management. Beyond simply tracking loaded datasets, the application organizes them into a structured Sample-Run-Dataset hierarchy, enabling users to define an analysis ‘context’ comprising hundreds of partial datasets common in serial crystallography. The system empowers users to operate on these groups collectively rather than as isolated files. It provides a context-sensitive interface for batch processing – launching pipelines like *XDS*, *xia2* or *autoPROC* (Vonrhein *et al.*, 2011 ▸) on multiple datasets simultaneously – and integrates downstream analysis tools for clustering, merging or solving structures directly from the selected context. This transforms the file list into an active workspace for high-throughput data management.

One unique feature of the iv is its ability to concurrently execute and visualize results from diverse data processing pipelines [*e.g.DOZOR*, *XDS*, *CrystFEL* (White *et al.*, 2012 ▸)] via a modular plugin architecture. Sharing a common base plot class, this system enables plugins to instantiate dedicated background workers (via *QThreadPool*) that process data streams in real time, whether triggered by live HDF5 file-writing events or replayed from existing datasets. The plugin interface resides within a collapsible dock but supports ‘peeling’ into independent floating windows for multi-monitor setups. Each plugin enables granular control over processing parameters through customized configuration dialogs (*e.g.* DozorSettingsDialog) and facilitates intelligent batching for full or partial dataset reprocessing. Results are ingested asynchronously via Redis streams or log-file parsing and visualized using *PyQtGraph*: global metrics (*e.g.* spot counts, resolution estimates, scale factors) are plotted against frame indices with interactive seek capabilities, while spatial results (spots, predictions, integration masks) are projected back onto the detector image via signal-slot mechanisms for immediate visual verification. This capability is important for the exploratory phase of serial crystallography.

Another important aspect of the iv is its support for beamline operations. It implements a set of tools to help users correct experiment metadata, such as beam center, detector distance *etc*. A beam center calibration module was developed to calibrate the beam center from ring diffraction patterns, such as those from powder standards or ice rings. On the basis of the known ring location, energy or detector distance can be calculated and checked against the beamline setup as part of the beamline setup process. A hot pixel finder utilizes statistical persistence analysis (high value with low variance) on random frame subsets to automatically identify stuck, dead or noisy pixels, assisting in updating the bad pixel mask used by the detector. Furthermore, it can prepare correct input files for third-party software often needed by users for manual data processing, such as *CrystFEL* or *HKL* (Otwinowski & Minor, 1997 ▸). Recognizing the massive storage footprint of serial crystallography, the viewer includes an HDF5 file management tool, the ‘Dataset Combiner’. This tool allows users to merge multiple datasets to create curated subsets (*e.g.* ‘Save Only Hits’) by filtering frames according to real-time quality metrics (*e.g.DOZOR* score) from the analysis stream, significantly reducing storage requirements.

### 2.3. Data processing pipelines

Various processing pipelines are available to users to process their data in real time or offline (Fig. 1 ▸). The core processing logic is encapsulated in modular pipelines tailored to specific software suites. These pipelines run as independent background processes, either locally or on a cluster via a *Slurm* job management system (Jette & Wickberg, 2023 ▸) as in the case of GM/CA, which deploys a Linux cluster of dozens of workstations with a shared BeeGFS file system and approximately 1500 CPU cores. Using the cluster facilitates faster data processing, but it is not a *QP2* software dependency. The job status is communicated through the Redis state database. This design allows flexibility in software development and testing, and facilitates integration of the pipelines with different modules within the package.

To provide rapid feedback during data collection or raster scans, we perform real-time spot finding using *DOZOR*, where per-frame processing completes in sub-second time. We implemented a wrapper for *DOZOR* that can launch concurrent processes on each data file, or batches of data files, to find Bragg spots and evaluate diffraction quality in near real time. The spots can be overlaid onto the diffraction image, and various metrics versus frame number can be displayed as 1D plots in the plugin window below the image. To mitigate false positives from ice rings or other artifacts, we enhanced the *DOZOR* analysis with a set of optional filters that can be applied to the spot list. These include (i) proximity rejection, where peaks falling within a minimum distance of each other are discarded; (ii) a ‘valid diffraction’ criterion, ensuring a minimum number of spots exist within a user-defined resolution shell (*e.g.* 15–4 Å); (iii) an automatic ice-ring removal routine that fits radial profiles to standard solvent models to exclude resolution bins deviating by more than 4.0 sigma; and (iv) geometric masking to exclude specific resolution bands or detector regions.

For quick characterization of one or a few initial assessment images, we added wrapper modules for *MOSFLM* and *XDS* to allow spot finding, indexing and strategy calculation for images collected at *e.g.* 0° and 90°. Following indexing, the Matthews coefficient (Winn *et al.*, 2011 ▸) can be calculated from the unit-cell dimensions and space group to estimate solvent content and the number of residues in the asymmetric unit (assuming ∼50% solvent content), aiding in phasing decisions. These can then be used directly, or combined with radiation absorption calculation, to set up data collection.

*GMCAPROC* is a locally developed Python wrapper around *XDS* that is optimized for concurrent data processing with data collection, processing partial or complete datasets. If more than one data series is present in a dataset, from a single crystal or multiple crystals, *XSCALE* (Kabsch, 2010 ▸) or *xia2.multiplex* (Gildea *et al.*, 2022 ▸) can be used to merge them. At the end of data processing, *GMCAPROC* can use a user-provided model, or automatically search the Protein Data Bank (https://www.wwpdb.org/) using the unit cell and space group to find a potential model, and use it to determine the crystal structure using *Dimple* (Winn *et al.*, 2011 ▸). This allows users to view the density map within minutes after data collection, particularly useful for high-throughput screening of ligands. Additionally, a module wrapping *xia2* and *autoPROC* is triggered at the end of collection to provide additional processing power for more challenging cases or to improve processing results. The *xia2* plugin also supports interactive multi-crystal merging via *xia2.multiplex*, which can be launched directly from the dataset context manager to combine datasets from multiple crystals collected in a single session.

**Figure 4:**
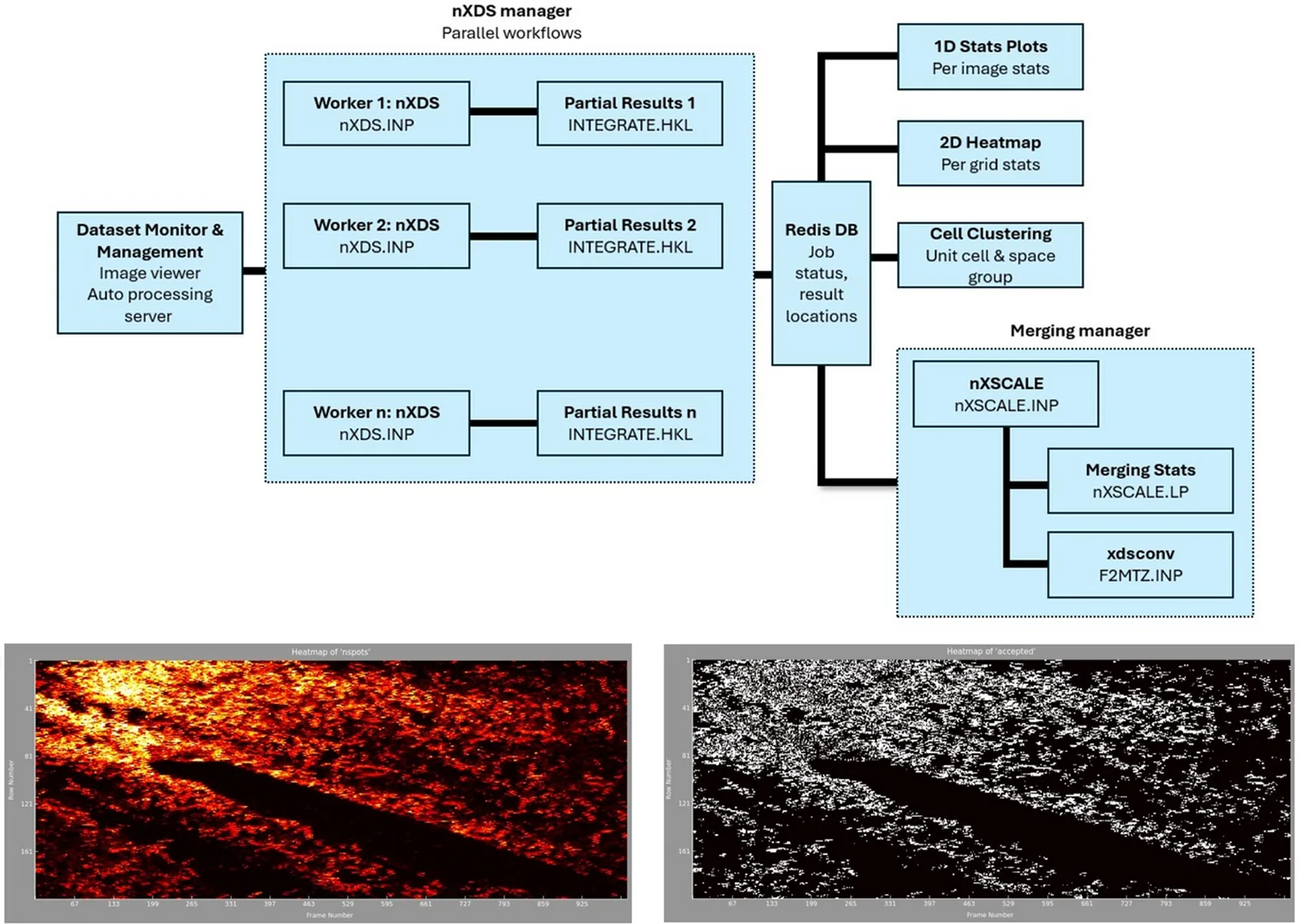
Schematic of the fixed target raster SSX processing pipeline using *nXDS*. (*a*) The *nXDSManager* partitions the dataset into independent jobs for parallel execution on a *Slurm* cluster, with partial results (INTEGRATE.HKL) collected from each worker. Results and job status are published to Redis for real-time visualization on the 2D heatmap of the grid and 1D plot of diffraction metrics versus frame index. A Merging Manager continuously accumulates partial results using *nXSCALE*, producing merged statistics and an mtz file via *xdsconv*, enabling on-the-fly assessment of overall data quality. (*b*) Representative grid maps generated by *nXDS*: (left) heatmap of diffraction spot counts across a 200 × 1000 grid (black: 0 spots; brightest: ≥1000 spots); (right) interactive grid map with successfully indexed images rendered as white cells. The grid was sampled in a row-wise serpentine pattern (1000 images per row; 0.01 s exposure; 5 µm beam; no attenuation) at room temperature using an EIGER X 16M detector at the GM/CA@APS 23-ID-B beamline. The indexing and integration of the dataset were completed in approximately 7 min using the GM/CA cluster, while the subsequent merging process took about 8 min using 128 CPU cores.

We implemented several pipelines for SSX, including *nXDS* (Kabsch, 2014 ▸), *CrystFEL* (White *et al.*, 2012 ▸) and *xia2.ssx* (Beilsten-Edmands *et al.*, 2024 ▸), to process large-scale datasets, particularly those from fixed-target raster scans. For such high-density experiments, *nXDS* is an excellent candidate for initial processing; its robustness and speed make it well suited for high-throughput workflows where initial characterization and rapid feedback are prioritized. We integrated these pipelines using a ‘scatter–gather’ architecture (Fig. 4 ▸), in which a central manager automatically partitions large datasets into smaller, independent processing jobs that are dispatched for concurrent execution via *Slurm*. This parallelization ensures that indexing and integration occur concurrently with data collection. Results such as spot counts, indexing rates and unit-cell parameters are aggregated in real time via Redis and projected onto the viewer’s heatmap. To facilitate the analysis of *nXDS* results, we implemented cell clustering algorithms using *DBSCAN* via *scikit-learn* (Ester *et al.*, 1996 ▸; Pedregosa *et al.*, 2011 ▸) or *NetworkX* (Hagberg *et al.*, 2008 ▸) to group unit cells from successfully indexed images. Additionally, an orientation analysis module computes pairwise misorientation angles between all indexed crystals, accounting for point-group symmetry, and clusters crystals with equivalent orientations to assess the degree of preferred orientation in the sample. While *nXDS* excels in speed, *xia2.ssx* and *CrystFEL* offer more extensive options, such as multi-lattice indexing, potentially leading to better results for complex datasets. For *CrystFEL*, we implemented automatic threshold estimation for *peakfinder8* (Barty *et al.*, 2014 ▸) based on robust statistics (median + 10× median absolute deviation, MAD) computed from the currently displayed image; the threshold can also be set manually when needed. To streamline parameter optimization, the settings dialog provides two interactive buttons: ‘Test Peaks’ runs peak finding on the current image and overlays the detected peaks for rapid validation, while ‘Test Index’ launches a lightweight indexing job to preview indexing performance on a representative frame before committing to full processing. These interactive diagnostics make parameter tuning more efficient for users during challenging SSX experiments.

To accommodate different user needs, the GM/CA processing system offers three distinct entry points to run the pipelines. The Automatic Processing Server acts as a background ‘watchdog’, automatically detecting new completed datasets (via Redis and file system events) and triggering standard pipelines without any user intervention. This provides a ‘hands-off’ experience, delivering initial results minutes after data collection ends. *DOZOR* is run for every image. For traditional (non-serial) data collection, *GMCAPROC* is run for 25%, 50% and 100% of the dataset, and *xia2* and *autoPROC* are run for 100% of the dataset. For raster-scan data collection, only *nXDS* is run for all images. For snapshots, *MOSFLM* and *XDS* are run to calculate index and strategy. For interactive experiments, Viewer-Triggered Processing allows users to trigger processing directly from the viewer. By continuously monitoring the current frame and view settings, the system allows users to initiate indexing or integration for the specific subset of data they are inspecting with a single click. For advanced users requiring granular control, the Manual Processing Dialog provides a comprehensive interface to configure all pipeline parameters (*e.g.* resolution cutoffs, space-group enforcement, unit-cell constraints) and resubmit jobs for specific datasets to the cluster. *QP2* adopts a robust resolution reporting strategy. The system prioritizes strict user-defined resolution cutoffs when provided. If automatic refinement is used, it falls back to an optimistic ‘high-resolution’ limit derived from the last statistically significant data shell (*e.g.* CC_1/2_ ∼ 0.35).

### 2.4. Processing Data Viewer and management

**Figure 5:**
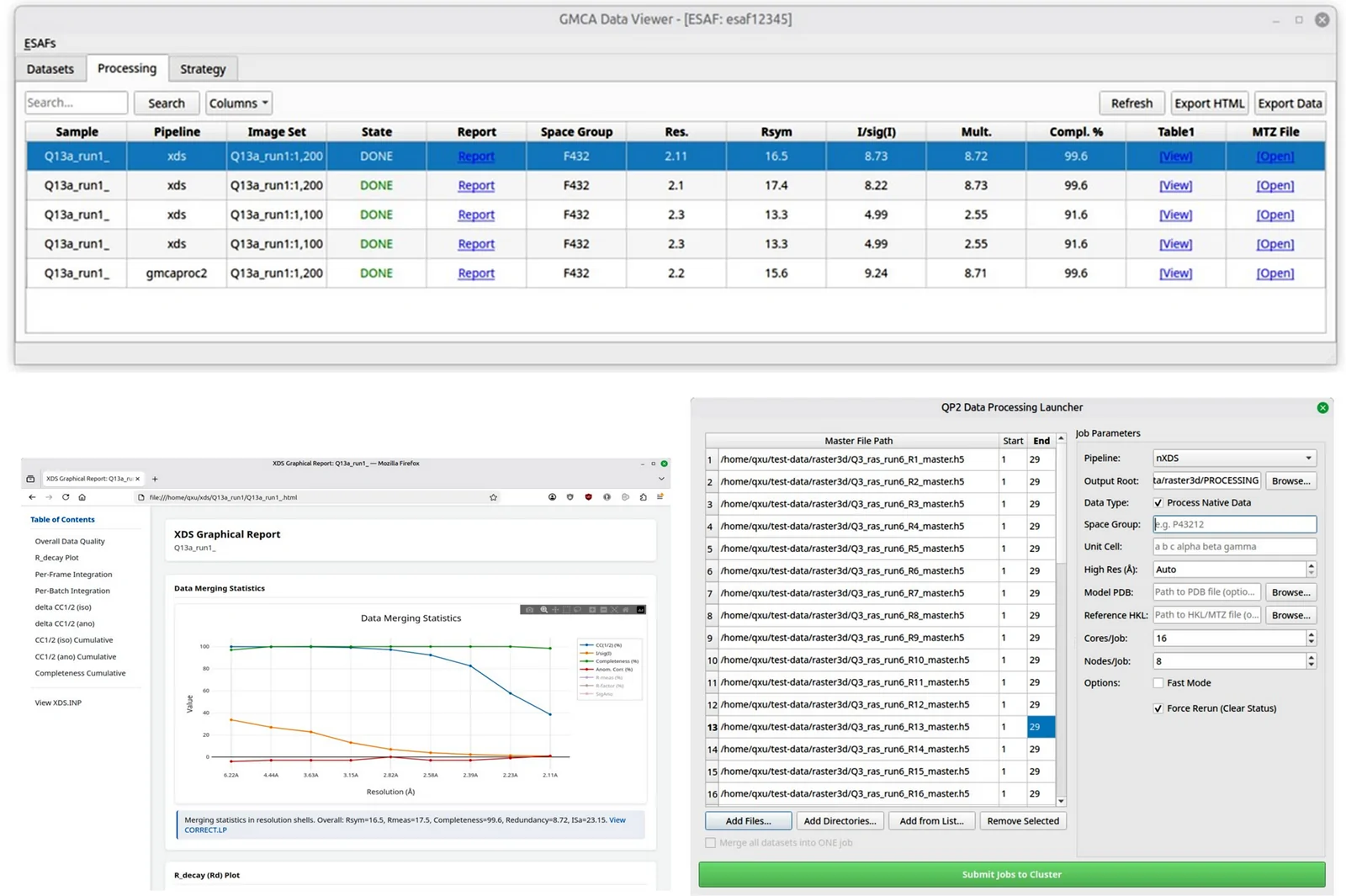
*QP2* Data Viewer and associated interfaces. (Top) The Processing tab of the Data Viewer displays a table of completed and running jobs, with columns for pipeline, image set, resolution, *R*_sym_, *I*/σ(*I*), multiplicity, completeness, and links to detailed HTML reports and mtz files. (Bottom left) An *XDS* Graphical Report opened directly from the table, showing data merging statistics including CC_1/2_, *I*/σ(*I*), and completeness as a function of resolution. (Bottom right) The *QP2* Manual Processing Launcher dialog, which enables users to submit custom reprocessing jobs with configurable pipeline, output directory, space group, resolution cutoff and cluster resource parameters.

Once the data are being processed, the job status and results are displayed in the Data Viewer (dv) (Fig. 5 ▸). It acts as the central dashboard for the experiment, providing a persistent, database-backed record of all data collected and processed. dv consists of three tabs: the Datasets tab tracks collected data as well as metadata about the crystal and data collection; the Processing tab tracks data processing status and results; and the Strategy tab tracks crystal screening and strategy calculation results. Every dataset, regardless of how it was collected, is tracked in the SQL database. The automatic processing server captures all datasets observed on the Redis stream, populating the dv with a complete history of the experiment. This persistence ensures full reproducibility: users can reload old datasets, review the parameters used for processing, and re-run analysis months or years later. The Processing tab offers a standard table view of the processing results, with shortcut links to open a visual summary of HTML reports or launch *Coot* (Emsley & Cowtan, 2004 ▸) to view electron-density maps if structure determination is successful. Beyond static history, the dv provides real-time monitoring of running jobs, allowing users to track the status of distributed processing tasks live as they execute on the computing cluster. This uniform and standardized output allows quick access to essential information and comparison of results from different pipelines.

### 2.5. Dose Planner

**Figure 6:**
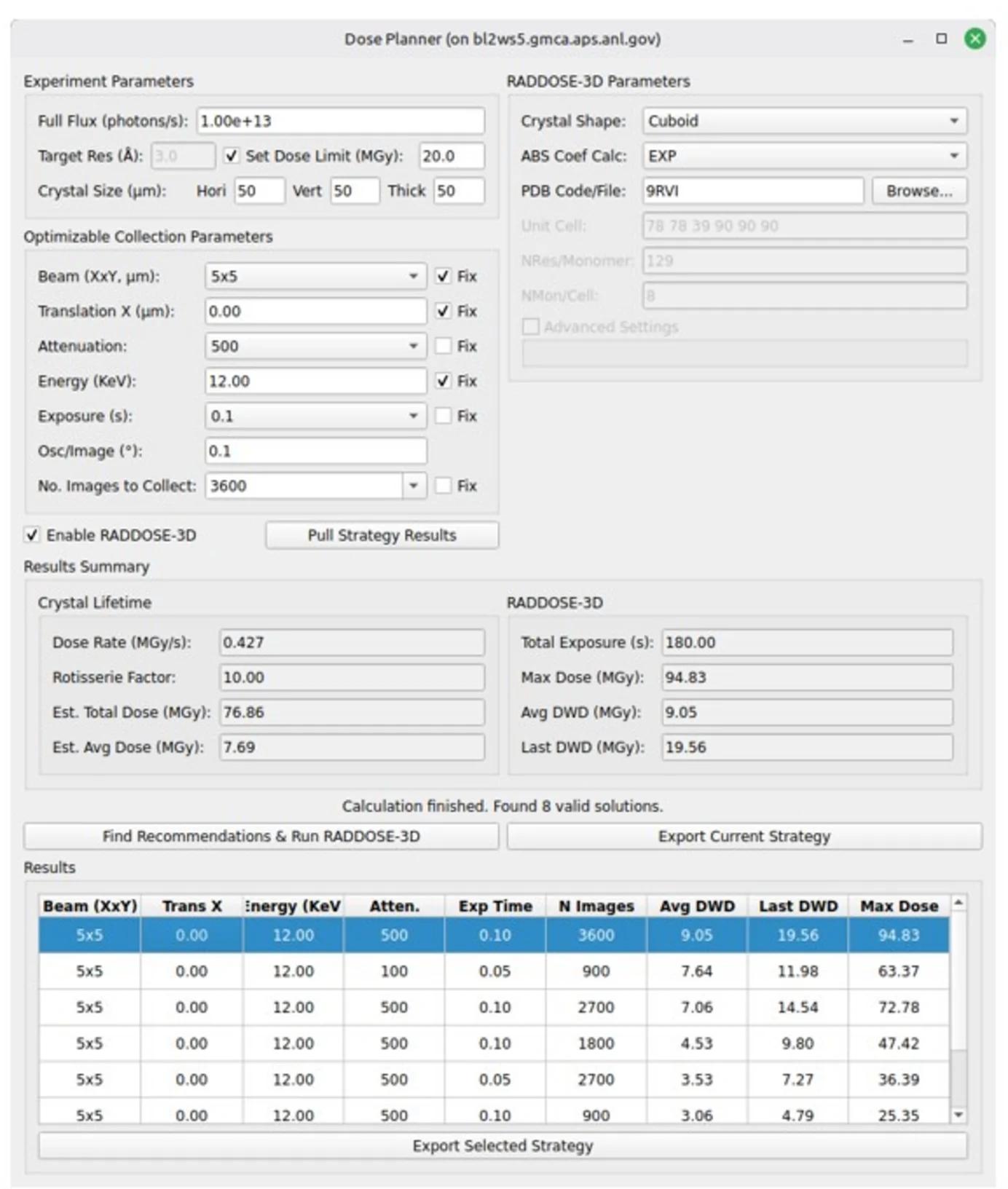
*QP2* Dose Planner interface. This module facilitates interactive radiation dose estimation and experiment strategy optimization. Users can simulate the expected dose for a given experiment condition or automatically determine optimal conditions – such as attenuation factors and exposure times – that maximize data collection efficiency within a user-defined dose limit.

With the adoption of high-flux microfocus beamlines, managing radiation damage has become a critical constraint in experiment design. *QP2* addresses this with an integrated Dose Planner (Fig. 6 ▸) that employs a hybrid two-stage optimization strategy to balance computational speed with simulation accuracy. The first stage utilizes an empirical model based on Holton’s formulation (Holton, 2009 ▸) to perform a rapid combinatorial sweep across experiment parameters (the size, flux at the sample position, attenuation and wavelength of the beam, and the exposure time). This pass filters for strategies that maximize collected frames within a user-defined dose limit (*e.g.* 30 MGy), applying a geometric ‘rotisserie factor’ to account for volume-averaged dose reduction during rotation. To verify these candidates, the system implements a ‘signature-based pruning’ algorithm that groups strategies with equivalent physical dose deposition profiles, minimizing redundant simulations. The resulting unique set is submitted to a parallelized *RADDOSE-3D* (Bury *et al.*, 2018 ▸) backend, which performs explicit pixel-by-pixel absorption calculations. This workflow provides users with a validated ‘diffraction weighted dose’ (DWD) metric. Furthermore, this dose optimization procedure is integrated with the strategy calculation module described earlier (*MOSFLM* and *XDS*). By feeding the optimized parameters into the automated data collection pipeline, *QP2* enables a closed-loop workflow where data collection strategies are dynamically tuned to maximize data quality while targeting the specified calculated dose limit.

### 2.6. The *Raster3D* pipeline

The *Raster3D* pipeline combines several tools in the package to analyze two orthogonal raster scans and generate recommendations for subsequent data collection. Building on the approximate 3D localization model and hotspot detection capabilities described in Section 2.2, it automates the workflow from diffraction analysis to collection planning. A collection tracker first identifies candidate raster pairs from consecutive collection events with the same base name and compatible scan modes; the pipeline worker then verifies that the two scans are approximately orthogonal from their omega angles. Once a valid pair is established, the pipeline proceeds through four stages: (i) polling for *DOZOR* or *nXDS* analysis results, with optional resubmission of missing jobs; (ii) reconstructing a 3D diffraction volume by combining the two 2D raster maps, followed by hotspot detection and PCA-based estimation of crystal size and orientation; (iii) running *XDS* and *MOSFLM* strategy calculations on the best crystal position to estimate indexing and collection parameters, including space group, unit cell, mosaicity, oscillation range and detector distance; and (iv) performing a dose-aware search over beam size, attenuation, exposure time and number of images, followed by *RADDOSE-3D* validation of the best candidate using crystal dimensions derived from the 3D reconstruction. Before reconstruction, a configurable quality gate can reject samples that do not show sufficient diffraction, on the basis of score, resolution and the number of strong frames. By default, the pipeline reports up to ten candidate crystal sites. For each accepted site, the output can include both voxel-space coordinates and motor-space centering positions, enabling direct transfer of the recommended target to beamline control software. For elongated crystals, the principal crystal axis is compared with the rotation axis: rods aligned with the rotation axis can be assigned to vector (helical) collection with defined start and end centering points, whereas rods with other orientations are treated with standard single-position collection. The pipeline also detects crystals that overlap along the rotation axis and applies a configurable policy to retain the strongest site, keep all sites or skip overlapping candidates.

### 2.7. Beamline integration and data archive

The *QP2* interface enables users to specify predefined crystallographic parameters, such as unit-cell dimensions, space group and known structural models. These parameters can be configured globally in the iv, while each individual pipeline provides its own settings dialog where users can override the global values. Alternatively, these parameters can be specified in the sample configuration spreadsheet, from which they are persisted by *QP2* from *PyBluIce* and automatically utilized by downstream data processing pipelines. *QP2* includes an integrated spreadsheet editor that provides a graphical interface for creating and managing these sample configuration files, supporting drag-and-drop sample re­arrangement, slot-by-slot crystal metadata entry (space group, path to a structural model, priority and crystal owner) and direct upload to the beamline server. *QP2* communicates processing results to the data acquisition control system (*PyBluIce*) via Redis, EPICS process variables or a REST API. This bidirectional integration allows, for example, optimized data collection strategies – calculated from initial characterization and optional dose optimization – to be exported directly back to *PyBluIce* for execution.

Each experiment is categorized under a uniform directory structure, defined by the APS Experimental Safety Assessment Form (ESAF) with a unique experiment ID. To ensure long-term data preservation and accessibility, *QP2* integrates with the APS Data Management system (Veseli *et al.*, 2018 ▸) through a dedicated automation module (dm_gmca). This module acts as an intelligent archiver, continuously scanning local high-performance storage (BeeGFS) for new experiment data. It automatically identifies valid experiment directories, retrieves associated scheduling metadata (Run Name, Experiment Date, ESAF ID *etc*.) from the APS central database and initiates secure data transfers to the facility’s archives for long-term storage. The system handles both raw diffraction images and downstream processing results, ensuring that the complete experiment record is preserved without requiring manual intervention from users or staff. The user can retrieve data from the APS Data Management system using the APS Data Globus endpoint (APS:DM:23ID) after the experiment is completed, or directly from GM/CA hosted Globus endpoints (GMCA 23ID/APS Data Collection, GMCA 23ID/APS Data Collection 2) with 6-month retention. This facility-specific feature would be changed when implementing *QP2* at a different facility.

### 2.8. AI assistant

To enhance user support and capabilities, we implemented an in-app AI assistant that functions in three distinct modes: as an interactive manual, a scripting copilot or a multi-user collaborative chat room. The module is built on a client–server architecture using OpenAI-compatible APIs, leveraging commercial LLMs accessed via Argonne’s Argo pr­oxy service – a facility-wide gateway that provides OpenAI-compatible access to a range of commercial models configurable by the user – and integrates a retrieval-augmented generation (RAG) system. A background worker indexes the *QP2* codebase and documentation, generating vector embeddings that are cached in Redis for rapid retrieval. This knowledge base allows the AI to query the software’s own source code to provide accurate, context-aware answers to user questions about features and usage. The assistant can also operate in an interactive ‘Chat-to-Code’ mode, accessing the application’s runtime namespace, including the active image viewer, data headers and graphics managers. Users can request complex actions in natural language – such as ‘mask all pixels above 10000 counts’ or ‘draw a resolution ring at 2.5 Å’ – and the assistant generates Python code to execute these tasks utilizing the internal API. Finally, chat data are synchronized via a Redis database, enabling a collaborative environment within the local beamline network where beamline staff can remotely assist users.

### 2.9. Implementation and development

Decoupling *QP2*’s data processing from the beamline control system offers significant architectural advantages. First, this separation minimizes dependencies, creating a self-contained package that is easily deployable across different environments, including the home laboratories of beamline users. Second, it isolates computationally intensive processing tasks from critical data acquisition loops, ensuring that high-rate data collection remains uninterrupted, even under heavy load. Finally, the event-driven architecture allows the Redis stream to be simulated using existing datasets. This capability enables offline testing and development without requiring valuable beamtime, facilitating continuous integration and robust validation of the pipeline.

While *QP2* evolved from the preceding *QP* framework, the codebase has been extensively rewritten and refactored through a collaborative process using LLMs, primarily Google Gemini and Claude. Leveraging the aforementioned ability to simulate the data stream offline, we decoupled UI development and algorithm validation from the physical beamline. This approach significantly accelerated development speed while ensuring strict operational correctness. Crucially, we found that domain expertise remains the cornerstone of successful implementation – providing architectural guidance, precise specifications, and the critical ability to validate outputs and detect hallucinations. LLMs proved exceptionally powerful tools for exploring alternative implementations and analyzing legacy logic, but they function best when paired with knowledgeable oversight. These experiences highlight the transformative potential of AI as a force multiplier in scientific software engineering. Active development encompasses new functional features, web interface enhancements and pipeline performance improvements, with additional support for remote serial data processing on Argonne National Laboratory’s high-performance computing clusters.

## 3. Software availability and portability

The core applications within *QuickProcess2* are optimized to operate seamlessly across both local and remote Linux desktop environments, as well as distributed high-performance computing (HPC) clusters. At GM/CA, users routinely conduct remote beamline sessions using *NoMachine* (*NX*), which provides direct access to these graphical tools over remote X-window connections. Although originally developed for the Linux environment at the GM/CA beamlines at the APS, components such as the image viewer and its data processing plugins can operate in standalone mode or on an institutional Linux cluster. To streamline deployment and eliminate the need for dedicated Redis and SQL servers, a custom module (sqlite_redis) was developed as a drop-in replacement for Redis. It utilizes shared database files on a distributed filesystem and is backed by the standard *SQLite* package. Additional third-party Python dependencies include *redis-py*, *SQLAlchemy*, *h5py*, *PyQt5*, *PyQtGraph*, *rcsb-api*, *opencv-python*, *scipy*, *scikit-learn*, *numpy*, *networkx*, *pandas* and *gemmi*. *QP2* is available as an open-source project. Source code, installation instructions and documentation are freely available at https://www.gmca.aps.anl.gov/computing/qp2 or https://doi.org/10.5281/zenodo.19262575.

## 4. Conclusion

*QP2* provides an integrated software platform for beamline operations, consolidating visualization, processing and experiment management into a single interface. By linking real-time visualization with automated metadata handling, the system facilitates data assessment during experiments. The architecture is designed to support both standard rotation datasets and large-scale serial crystallography projects, offering a unified tool for diverse data collection strategies.

## References

[bb1] Adams, P. D., Afonine, P. V., Bunkóczi, G., Chen, V. B., Davis, I. W., Echols, N., Headd, J. J., Hung, L.-W., Kapral, G. J., Grosse-Kunstleve, R. W., McCoy, A. J., Moriarty, N. W., Oeffner, R., Read, R. J., Richardson, D. C., Richardson, J. S., Terwilliger, T. C. & Zwart, P. H. (2010). *Acta Cryst.* D**66**, 213–221. 10.1107/S0907444909052925 PMC2815670 20124702

[bb2] Arvai, A. (2012). *adxv*, https://www.scripps.edu/tainer/arvai/adxv.html.

[bb3] Barty, A., Kirian, R. A., Maia, F. R. N. C., Hantke, M., Yoon, C. H., White, T. A. & Chapman, H. (2014). *J. Appl. Cryst.* **47**, 1118–1131. 10.1107/S1600576714007626 PMC4038800 24904246

[bb4] Beilsten-Edmands, J., Parkhurst, J. M., Winter, G. & Evans, G. (2024). *Methods Enzymol.* **709**, 207–244. 10.1016/bs.mie.2024.10.004 39608945

[bb5] Bury, C. S., Brooks–Bartlett, J. C., Walsh, S. P. & Garman, E. F. (2018). *Protein Sci.* **27**, 217–228. 10.1002/pro.3302 PMC5734275 28921782

[bb6] Delagenière, S., Brenchereau, P., Launer, L., Ashton, A. W., Leal, R., Veyrier, S., Gabadinho, J., Gordon, E. J., Jones, S. D., Levik, K. E., McSweeney, S., Monaco, S., Nanao, M., Spruce, D., Svensson, O., Walsh, M. A. & Leonard, G. A. (2011). *Bioinformatics* **27**, 3186–3192. 10.1093/bioinformatics/btr535 21949273

[bb7] Emsley, P. & Cowtan, K. (2004). *Acta Cryst.* D**60**, 2126–2132. 10.1107/S0907444904019158 15572765

[bb8] Ester, M., Kriegel, H.-P., Sander, J. & Xu, X. (1996). *Proceedings of the 2nd International Conference on Knowledge Discovery and Data Mining (KDD’96)*, pp. 226–231. Association for the Advancement of Artificial Intelligence.

[bb9] Fisher, S. J., Levik, K. E., Williams, M. A., Ashton, A. W. & McAuley, K. E. (2015). *J. Appl. Cryst.* **48**, 927–932. 10.1107/S1600576715004847 PMC4453979 26089766

[bb10] Fodje, M., Janzen, K., Berg, R., Black, G., Labiuk, S., Gorin, J. & Grochulski, P. (2012). *J. Synchrotron Rad.* **19**, 274–280. 10.1107/S0909049511056305 22338690

[bb11] Gildea, R. J., Beilsten-Edmands, J., Axford, D., Horrell, S., Aller, P., Sandy, J., Sanchez-Weatherby, J., Owen, C. D., Lukacik, P., Strain-Damerell, C., Owen, R. L., Walsh, M. A. & Winter, G. (2022). *Acta Cryst.* D**78**, 752–769. 10.1107/S2059798322004399 PMC9159281 35647922

[bb12] Hagberg, A. A., Schult, D. A. & Swart, P. J. (2008). *Proceedings of the 7th Python in Science Conference (SciPy 2008)*, pp. 11–15.

[bb13] Holton, J. M. (2009). *J. Synchrotron Rad.* **16**, 133–142.

[bb14] Jette, M. A. & Wickberg, T. (2023). Vol. 14283, *Job Scheduling Strategies for Parallel Processing*, edited by D. Klusáček, J. Corbalán & G. P. Rodrigo, pp. 3–23. Cham: Springer.

[bb15] Kabsch, W. (2010). *Acta Cryst.* D**66**, 125–132. 10.1107/S0907444909047337 PMC2815665 20124692

[bb16] Kabsch, W. (2014). *Acta Cryst.* D**70**, 2204–2216. 10.1107/S1399004714013534 PMC4118830 25084339

[bb17] Leslie, A. G. W. & Powell, H. R. (2007). *Evolving Methods for Macromolecular Crystallography*, Vol. 245, pp. 41–51. Springer.

[bb18] Moore, O., Jessurun, N., Chase, M., Nemitz, N. & Campagnola, L. (2023). *Proceedings of the 22nd Python in Science Conference (SciPy 2023)*.

[bb19] Otwinowski, Z. & Minor, W. (1997). *Methods Enzymol.* **276**, 307–326. 10.1016/S0076-6879(97)76066-X 27754618

[bb20] Pedregosa, F., Varoquaux, G., Gramfort, A., Michel, V., Thirion, B., Grisel, O., Blondel, M., Prettenhofer, P., Weiss, R. & Dubourg, V. (2011). *J. Mach. Learn. Res.* **12**, 2825–2830.

[bb22] Pothineni, S. B., Venugopalan, N., Ogata, C. M., Hilgart, M. C., Stepanov, S., Sanishvili, R., Becker, M., Winter, G., Sauter, N. K., Smith, J. L. & Fischetti, R. F. (2014). *J. Appl. Cryst.* **47**, 1992–1999. 10.1107/S1600576714022730 PMC4248568 25484844

[bb24] Sheldrick, G. M. (2008). *Acta Cryst.* A**64**, 112–122. 10.1107/S0108767307043930 18156677

[bb25] van der Walt, S., Schönberger, J. L., Nunez-Iglesias, J., Boulogne, F., Warner, J. D., Yager, N., Gouillart, E. & Yu, T. (2014). *PeerJ* **2**, e453. 10.7717/peerj.453 PMC4081273 25024921

[bb26] Veseli, S., Schwarz, N. & Schmitz, C. (2018). *J. Synchrotron Rad.* **25**, 1574–1580. 10.1107/S1600577518010056 PMC6140395 30179199

[bb27] Virtanen, P., Gommers, R., Oliphant, T. E., Haberland, M., Reddy, T., Cournapeau, D., Burovski, E., Peterson, P., Weckesser, W., Bright, J., van der Walt, S. J., Brett, M., Wilson, J., Millman, K. J., Mayorov, N., Nelson, A. R. J., Jones, E., Kern, R., Larson, E., Carey, C. J., Polat, İ., Feng, Y., Moore, E. W., VanderPlas, J., Laxalde, D., Perktold, J., Cimrman, R., Henriksen, I., Quintero, E. A., Harris, C. R., Archibald, A. M., Ribeiro, A. H., Pedregosa, F., van Mulbregt, P., Vijaykumar, A., Bardelli, A. P., Rothberg, A., Hilboll, A., Kloeckner, A., Scopatz, A., Lee, A., Rokem, A., Woods, C. N., Fulton, C., Masson, C., Häggström, C., Fitzgerald, C., Nicholson, D. A., Hagen, D. R., Pasechnik, D. V., Olivetti, E., Martin, E., Wieser, E., Silva, F., Lenders, F., Wilhelm, F., Young, G., Price, G. A., Ingold, G., Allen, G. E., Lee, G. R., Audren, H., Probst, I., Dietrich, J. P., Silterra, J., Webber, J. T., Slavič, J., Nothman, J., Buchner, J., Kulick, J., Schönberger, J. L., de Miranda Cardoso, J. V., Reimer, J., Harrington, J., Rodríguez, J. L. C., Nunez-Iglesias, J., Kuczynski, J., Tritz, K., Thoma, M., Newville, M., Kümmerer, M., Bolingbroke, M., Tartre, M., Pak, M., Smith, N. J., Nowaczyk, N., Shebanov, N., Pavlyk, O., Brodtkorb, P. A., Lee, P., McGibbon, R. T., Feldbauer, R., Lewis, S., Tygier, S., Sievert, S., Vigna, S., Peterson, S., More, S., Pudlik, T., Oshima, T., Pingel, T. J., Robitaille, T. P., Spura, T., Jones, T. R., Cera, T., Leslie, T., Zito, T., Krauss, T., Upadhyay, U., Halchenko, Y. O. & Vázquez-Baeza, Y. (2020). *Nat. Methods* **17**, 261–272.

[bb28] Vonrhein, C., Flensburg, C., Keller, P., Sharff, A., Smart, O., Paciorek, W., Womack, T. & Bricogne, G. (2011). *Acta Cryst.* D**67**, 293–302. 10.1107/S0907444911007773 PMC3069744 21460447

[bb29] White, T. A., Kirian, R. A., Martin, A. V., Aquila, A., Nass, K., Barty, A. & Chapman, H. N. (2012). *J. Appl. Cryst.* **45**, 335–341.

[bb30] Winn, M. D., Ballard, C. C., Cowtan, K. D., Dodson, E. J., Emsley, P., Evans, P. R., Keegan, R. M., Krissinel, E. B., Leslie, A. G. W., McCoy, A., McNicholas, S. J., Murshudov, G. N., Pannu, N. S., Potterton, E. A., Powell, H. R., Read, R. J., Vagin, A. & Wilson, K. S. (2011). *Acta Cryst.* D**67**, 235–242. 10.1107/S0907444910045749 PMC3069738 21460441

[bb31] Winter, G. (2010). *J. Appl. Cryst.* **43**, 186–190.

[bb32] Winter, G., Waterman, D. G., Parkhurst, J. M., Brewster, A. S., Gildea, R. J., Gerstel, M., Fuentes-Montero, L., Vollmar, M., Michels-Clark, T., Young, I. D., Sauter, N. K. & Evans, G. (2018). *Acta Cryst.* D**74**, 85–97. 10.1107/S2059798317017235 PMC5947772 29533234

[bb33] Zander, U., Bourenkov, G., Popov, A. N., de Sanctis, D., Svensson, O., McCarthy, A. A., Round, E., Gordeliy, V., Mueller-Dieckmann, C. & Leonard, G. A. (2015). *Acta Cryst.* D**71**, 2328–2343. 10.1107/S1399004715017927 PMC4631482 26527148

